# Palbociclib plus endocrine therapy in hormone receptor-positive and HER2 negative metastatic breast cancer: a multicenter real-world study in the northwest of China

**DOI:** 10.1186/s12885-023-10568-0

**Published:** 2023-01-30

**Authors:** Jiao Yang, Bing Zhao, Xiaoling Ling, Donghui Li, Jiuda Zhao, Yonggang Lv, Guangxi Wang, Xinlan Liu, Nanlin Li, Jin Yang

**Affiliations:** 1 Departments of Medical Oncology, The First Affiliated Hospital of Xi’an Jiaotong University, 277 West Yanta Road, 710061 Xi’an, Shaanxi P.R. China; 2 Breast Internal Medicine Department, The 3rd Affiliated Teaching Hospital of XinJiang Medical University (Affiliated Tumor Hospital), No. 789 Suzhou East Road, Xinshi District, 830011 Urumqi, Xinjiang China; 3 Department of Oncology, The First Hospital of Lanzhou University, Lanzhou University, 730000 Lanzhou, Gansu China; 4 Department of Medical Oncology, Shaanxi Provincial People’s Hospital, 710068 Xi’an, Shaanxi China; 5 Breast Disease Diagnosis and Treatment Center of Affiliated Hospital of Qinghai University & Affiliated Cancer Hospital of Qinghai University, Xining, China; 6 Department of Thyroid Breast Surgery, the Affiliated Hospital of Northwest University, Xi’an No.3 Hospital, Xi’an, Shaanxi China; 7 Department of Oncology, General Hospital of Ningxia Medical University, 750004 Yinchuan, Ningxia China; 8 Department of Thyroid, Breast and Vascular Surgery, Xijing Hospital, The Fourth Military Medical University, 710032 Xi’an, China; 9 Department of Medical Oncology, Shaanxi Provincial Tumor Hospital, Xi’an, China

**Keywords:** Metastatic breast cancer, CDK4/6 inhibitor, Endocrine therapy, Real-world

## Abstract

**Background:**

Real-world data of Palbociclib are insufficient in China. This study aimed to investigate the treatment pattern and real-world outcomes in hormone receptor positive and human epidermal growth factor 2 receptor negative (HR+/HER2-) metastatic breast cancer (MBC) patients treated with Palbociclib in the northwest of China.

**Methods:**

HR+/HER2- MBC patients who received Palbociclib in 8 centers from July 2017 to September 2019 were retrospectively included in this study. Real-world objective response rate (ORR), progression-free survival (PFS) and safety profiles were analyzed. The survival curves were plotted by the Kaplan-Meier method to analyze PFS, which was verified by the log-rank test.

**Results:**

In total, 211 women were eligible for the analysis. A total of 85 patients (40.3%), 78 (37.0%), and 48 (22.7%) received Palbociclib in the first-, second-, third- or later-line setting, respectively. 46 patients achieved partial response and 145 patients experienced stable disease, with an ORR of 21.8% and a disease control rate of 90.5%. Following a median follow-up period of 14.2 months, the median PFS was 12.2 months (95% confidence interval, 10.1-14.3 m), and the median overall survival was not reached. Early Palbociclib initiation, sensitivity or acquired resistance to endocrine therapy, estrogen receptor and progesterone receptor double positivity, less than 3 metastatic sites, without visceral metastasis, bone metastasis only, without prior chemotherapy or endocrine therapy were associated with a prolonged PFS in MBC (All P < 0.05). The most common grade 3 or 4 adverse events (AE) was neutropenia (36.5%), and the most common nonhematologic AE was fatigue (10.9%). No patient experienced AE leading to treatment discontinuation.

**Conclusion:**

Palbociclib plus endocrine therapy exhibited favorable effectiveness and manageable toxicities in the real-world setting, supporting their use in Chinese patients with HR+/HER2 − MBC.

**Supplementary Information:**

The online version contains supplementary material available at 10.1186/s12885-023-10568-0.

## Introduction

Breast cancer accounts for 30% of all newly diagnosed cancers in women worldwide, with 2.81 million cases reported in 2021, of which 45.4% are Asian patients [1]. Approximately 70% of breast cancer cases are hormone receptor positive and human epidermal growth factor receptor 2 (HR+/HER2-). For more than a decade, endocrine therapy (ET) has been the primary choice for locally advanced or metastatic breast cancer (MBC) with HR+/HER2- that is not life-threatening [2]. Nevertheless, it is inevitable that endocrine resistance will appear through diverse mechanisms. Therefore, efforts have been made to unearth the mechanism of resistance, develop innovative methods to overcome primary or acquired endocrine resistance and delay the use of chemotherapy, thus improving patients’ outcomes and quality of life [3]. With a better understanding of breast cancer biology and endocrine resistance mechanisms, cyclin-dependent kinase 4/6 inhibitors (CDK4/6i) have been developed, which is clinically effective and tolerable when combined with ET [4]. On the basis of multiple randomized controlled trials, current guidelines recommend the addition of CDK4/6i (Abemaciclib, Ribociclib, or Palbociclib) to ET as the standard care for HR+/HER2- MBC [5, 6].

Palbociclib, an orally bioavailable CDK4/6i, has been proved to induce cell-cycle arrest in endocrine-resistant breast cancer cell lines and to have synergistic anti-tumor effects in preclinical studies [7]. After receiving accelerated approval from the US Food and Drug Administration for use in combination with ET to treat HR+/HER2- MBC in 2015 based on progression-free survival (PFS) evidence from PALOMA studies, Palbociclib became available in China in July 2018 [8–10]. Despite this, Palbociclib’s translation to daily practice can sometimes be challenging, since its effects aren’t exactly the same as those shown in randomized clinical trials. In the most obvious sense, PALOMA trials cannot fully represent clinical patients according to their rigorous inclusion criteria, such as the higher proportion of postmenopausal women in western population included. Subgroup analyses of Asian patients revealed similar survival outcomes and quality of life, but with more hematologic toxicity [11]. However, Asian populations and premenopausal women were under-represented with proportions of only 10-30% and 17-21% in the trials, respectively, as well as the proportions of patients with young age, specific metastatic site, different treatment lines, failure to prior endocrine therapy or chemotherapy, diverse sensitive degree to endocrine therapy and so on. In addition, compared to the western population, Chinese patients had a higher proportion of premenopausal women, more first-line chemotherapy instead of ET, and more patients experienced visceral metastasis. How to optimize the individual therapy remains a key issue. Therefore, real-world studies are becoming increasingly important in answering questions commonly encountered in clinical practice that not revealed by randomized trials.

Several real-world analyses of Palbociclib have shown similar effectiveness and safety as those observed in the clinical trials. However, they were limited by small sample sizes and/or short follow-up periods [12–14]. To our knowledge, real-world data is insufficient on Palbociclib from Chinese patients, even though there were three studies conducted in Asian patients [15–17]. Thus, this multicenter study was conduct to determine the real-world treatment pattern and outcome of Palbociclib plus ET in unselected MBC women from the Northwest of China.

## Methods

### Study design and patients

In this real-world study conducted in 8 centers from northwest China, patients who met the inclusion and exclusion criteria from July 2017 to September 2019 were retrospectively selected. Eligibility criteria were as follows: (1) Patients with HR+/HER2- MBC receiving at least two cycles of Palbociclib plus ET; (2) Receiving at least one tumor assessment based on Response Evaluation Criteria in Solid Tumors version 1.1 (RECISTv1.1). Patients with other primary malignancies or those lost to follow-up were excluded. A total of 211 patients were finally selected from eight centers, including the First Affiliated Hospital of Xi’an Jiao Tong University (Shaanxi, China), Xijing Hospital (Shaanxi, China), Shaanxi Provincial People’s Hospital (Shaanxi, China), Affiliated Hospital of Northwest University (Shaanxi, China), Affiliated Tumor Hospital of Xinjiang Medical University (Xinjiang, China), General Hospital of Ningxia Medical University (Ningxia, China), First Affiliated Hospital of Lanzhou University (Gansu, China), Affiliated Hospital of Qinghai University (Qinghai, China).

The study was conducted according to the Declaration of Helsinki (as revised in 2013) and was approved by the Ethics Committee of the First Affiliated Hospital of Xi’an Jiao Tong University. Informed consent was obtained from all the patients or their legal guardians.

### Data collection

Clinical characteristics of the patients were collected from medical records, including age, ethnicity, menopausal status, Eastern Cooperative Oncology Group (ECOG) performance status (PS), and metastatic sites at the initiation of Palbociclib. Prior therapy including chemotherapy and ET adopted in the neoadjuvant, adjuvant and metastatic setting was recorded. Treatment pattern was captured, including the treatment lines, starting dose, dose modifications and combination regimens. Follow-up was achieved via outpatient review or inpatient examination, and conducted every eight weeks using medical records. Follow-up was due by November 13th 2021.

The stage of patients was determined based on clinical, radiographic, and pathological findings according to the eighth edition of the American Joint Committee on Cancer (AJCC) tumor-node-metastasis (TNM) staging system [18]. MBC was defined as locally advanced, which was unresectable, or metastatic. A rate of nuclear staining of ≥ 1% using immunohistochemistry (IHC) was defined as estrogen receptor (ER) or progesterone receptor (PgR) positive, and patient who showed ER or PgR positive was considered hormone receptor positive [19]. Patients with an IHC staining score of 2 and with the HER2 gene amplification as determined by flourescence in situ hybridization, or an IHC staining score of 3 were considered HER2 positive [20]. Menopause was defined as the cessation of menstruation permanently, including natural menopause and artificial menopause.

Radiology and pathology reports were used to determine the baseline metastasis status of patients. Metastases types included local metastasis, bone metastasis only, and visceral metastasis with or without bone metastasis. Primary resistance to ET was identified if disease progression occurred within 2 years after adjuvant ET or within 6 months during first-line ET in metastatic setting, according to the 5th International Consensus Conference for Advanced Breast Cancer (ABC5) [5]. Secondary endocrine resistance is defined as relapse while on adjuvant ET but after the first 2 years, or relapse within 12 months of completing adjuvant ET, or PD ≥ 6 months after initiating ET for ABC, while on ET. All the patients were assessed according to sensitivity to their initial endocrine therapy, namely, adjuvant ET or first line ET, whichever comes first.

### Endpoints

The primary endpoint was PFS, which was defined as the time from Palbociclib initiation to disease progression or death, whichever came first. The 6- and 12-month PFS rates were calculated according to the Kaplan–Meier method, and survival rates were compared by log-rank test. The secondary endpoints included the objective response rate (ORR) and disease control rate (DCR). ORR was defined as the percentage of patients achieved complete response (CR) or partial response (PR). DCR was defined as the percentage of CR, PR, and stable disease (SD). Subgroup analysis were conducted according to treatment lines (received Palbociclib plus ET as first-line, second-line, or later-line treatment).

All adverse events (AE) during the treatment were recorded, and the severity of AEs was graded according to the Common Terminology Criteria for Adverse Events version 4.0 (CTCAE 4.0). Palbociclib comes in three different dosage forms, including 125, 100, and 75 mg. The recommended starting dosage is 125 mg (once daily for three weeks, then one week off). Patients were allowed to adjust treatment dosage and cycles or to discontinue treatment based on the safety profiles.

### Statistical analyses

Descriptive statistics were used for all the variables of interest. Continuous variables were presented as median and range, and categorical variables were presented as numbers and percentages. A correlation analysis between patient characteristics and tumor response was conducted using the Pearson’s chi-square test or Fisher’s exact test. The Kaplan-Meier method was used to plot the survival curve, and the log-rank test was used to assess differences between subgroups. The univariable Cox proportional hazards model was used to estimate hazard ratios (HR) and 95% confidence interval (CI) of PFS. A two-sided p-value of less than 0.05 was considered statistically significant. SPSS software (version 21.0, IBM Inc., Chicago, IL) and GraphPad Prism (version 8, GraphPad Software, San Diego, CA, US) were used for statistical analysis and graphics.

## Results

### Patient characteristics

**Fig. 1:**
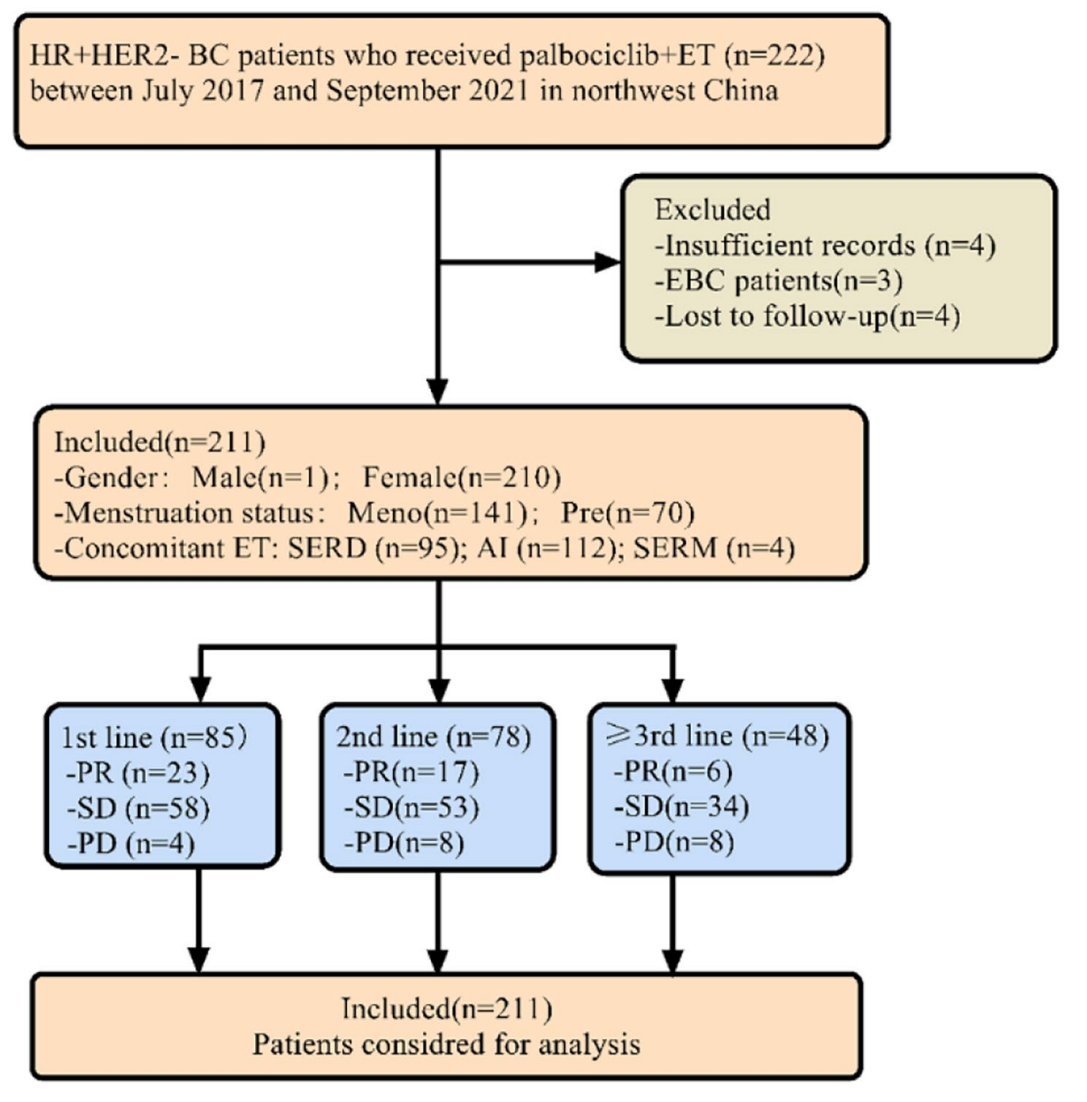
Flow chart of the study. HR+/HER2-, Hormone receptor-positive and Human epidermal growth factor receptor 2 negative; ET, Endocrine therapy; EBC, Early breast cancer; Meno, Menopausal status; Pre, Premenopausal status; SERD, Selective estrogen receptor degrader; AI, Aromatase inhibitor; SERM, Selective estrogen receptor modulator; PR, Partial response; SD, Stable disease; PD, Progressive disease

A total of 222 MBC patients treated with Palbociclib was enrolled in the study between July 2017 and September 2019. Among them, 11 patients were excluded due to lost records, early stage or lost follow up, and 211 patients were included in the final analysis (Fig. 1). The median age was 53 (range 29 to 88) years. Approximately two thirds of patients (67.1%) were menopausal, and most had an ECOG of 0–1 (96.2%). 50 patients (23.7%) were initially diagnosed as de novo stage IV. Majority of tumors were either sensitive (94, 44.6%) or acquired resistant (92, 43.6%) to recent ET, with the remainder being primary resistant (25, 11.8%). A total of 134 patients (63.5%) had visceral metastases, while 42 (19.9%) had bone metastases only. One third of patients developed metastases involving three or more organs. 85 (40.3%), 78 (37.0%) and 48 (22.7%) patients received Palbociclib as the first-, second-, third or later-line treatment, respectively. The concomitant ET regimen was most likely to be aromatase inhibitors (AI, 53.1%), followed by selective estrogen receptor degrader (SERD, 45.0%) and selective estrogen receptor modulators (SERM, 1.9%) (Table 1).

**Table 1:** Baseline characteristics of patients in different treatment-line settings

**Characteristic**	**All patients(n = 211)**	**1st line(n = 85)**	**2nd line(n = 78)**	**≥ 3rd line(n = 48)**
**Median age, years (range)**	53(29–88)	55(29–84)	51(32–88)	53(29–57)
≥65y	41(19.4)	18(21.2)	17(21.8)	6(12.5)
<65y	170(80.6)	67(78.8)	61(78.2)	42(87.5)
**Menstruation status**				
Menopausal	141(67.1)	58(69.0)	53(67.9)	30(62.5)
Premenopausal	70(32.9)	27(31.0)	25(32.1)	18(37.5)
**ECOG PS**				
0	73(34.6)	29(34.1)	30(38.5)	14(29.1)
1	130(61.6)	53(62.4)	45(57.7)	32(66.7)
2	8(3.8)	3(3.5)	3(3.8)	2(4.2)
**IV stage of first diagnosis**				
Yes	50(23.7)	27(31.8)	12(15.4)	11(22.9)
No	161(76.3)	58(68.2)	66(84.6)	37(77.1)
**Hormone receptor status**				
ER + PgR+	165(78.2)	71(83.5)	57(73.1)	37(77.1)
ER + PgR-	46(21.8)	14(16.5)	21(26.9)	11(22.9)
**ER status**				
≥ 50%	182(86.3)	70(82.4)	70(89.7)	42(87.5)
<50%	29(13.7)	15(17.6)	8(10.3)	6(12.5)
**Sensitivity to ET**				
Sensitivity	94(44.6)	53(62.4)	26(33.3)	15(31.3)
Acquired resistance	92(43.6)	28(32.9)	39(50.0)	25(52.0)
Primary resistance	25(11.8)	4(4.7)	13(16.7)	8(16.7)
**Number of metastatic sites**				
≥3	69(32.7)	20(23.5)	28(35.9)	21(43.7)
<3	142(67.3)	65(76.5)	50(64.1)	27(56.3)
**Presence of visceral metastasis**				
Yes	134(63.5)	44(51.8)	52(66.7)	38(79.2)
No	77(36.5)	41(48.2)	26(33.3)	10(20.8)
**Metastatic sites**				
Bone only	42(19.9)	20(23.5)	16(20.5)	6(12.5)
Lung involvement	72(34.1)	25(29.4)	27(34.6)	20(41.7)
Liver involvement	78(37.0)	24(28.2)	29(37.2)	25(52.1)
Brain involvement	15(7.1)	3(3.5)	6(7.7)	6(12.6)
Bone marrow	3(1.4)	2(2.4)	1(1.3)	-
**Concomitant ET**				
FULV	95(45.0)	35(41.2)	43(55.1)	17(35.4)
AI	112(53.1)	47(55.3)	34(43.6)	31(64.6)
TAM/TORE	4(1.9)	3(3.5)	1(1.3)	-

Values are presented as number (%) unless otherwise indicated. ECOG, Eastern Cooperative Oncology Group; ER, Estrogen receptor; PgR, Progesterone receptor; ET, Endocrine therapy; FULV, Fulvestrant; AI, Aromatase Inhibitors; TAM, Tamoxifen; TORE, Toremifene

### Prior therapy

In terms of prior therapy, 173 (82%) of patients had received radical surgery in the early stages of their disease, followed by chemotherapy, ET and radiotherapy in 139 (65.9%), 128 (60.7%) and 98 (46.4%) of cases, respectively. The adjuvant ET agent was more likely to be SERM (51.6%) instead of AI (48.4%). A total of 82 (38.9%) and 63 (29.9%) patients received prior chemotherapy and ET as palliative therapy, respectively. The most common palliative endocrine regimen was fulvestrant (58.7%), followed by AI (34.9%) and SERM (6.4%) (Table 2).

**Table 2:** Prior therapy for early breast cancer and metastatic breast cancer

**Prior therapy for EBC**	**All patients(n = 211)**	**1st line(n = 85)**	**2nd line(n = 78)**	**≥ 3rd line(n = 48)**
Neoadjuvant CHT	36(17.1)	17(20.0)	14(17.9)	5(10.4)
Surgery	173(82.0)	62(72.9)	69(88.5)	42(87.5)
Adjuvant CHT	139(65.9)	51(60)	49(62.8)	39(81.3)
Adjuvant RT	98(46.4)	37(43.5)	35(44.9)	26(54.2)
Adjuvant ET	128(60.7)	45(52.9)	48(62.8)	34(70.8)
SERM	66(51.6)	22(47.8)	25(52.1)	19(55.9)
AI	62(48.4)	24(52.2)	23(47.9)	15(44.1)
*Letrozole*	35(27.3)	13(28.3)	10(20.8)	12(35.2)
*Anatrozole*	5(3.9)	2(4.3)	1(2.2)	2(5.9)
*Exemestane*	13(10.2)	4(8.7)	8(16.7)	1(2.9)
*NA*	9(7.0)	5(10.9)	4(8.7)	-
**Prior therapy for MBC**				
Prior CHT for MBC	82(38.9)	7(8.2)	38(48.7)	37(77.1)
Prior ET for MBC	63(29.9)	-	30(38.5)	33(68.8)
SERM	4(6.3)	-	3(10.0)	1(3.0)
AI	22(34.9)	-	12(40.0)	10(30.3)
SERD	37(58.7)	-	15(50.0)	22(66.7)

Values are presented as number (%) unless otherwise indicated. EBC, Early breast cancer; CHT, Chemotherapy; RT, Radiation therapy; ET, Endocrine therapy; SERM, Selective estrogen receptor modulators; AI, Aromatase inhibitors; MBC, Metastatic breast cancer; SERD, Selective estrogen receptor degrader

### Treatment effectiveness

**Fig. 2:**
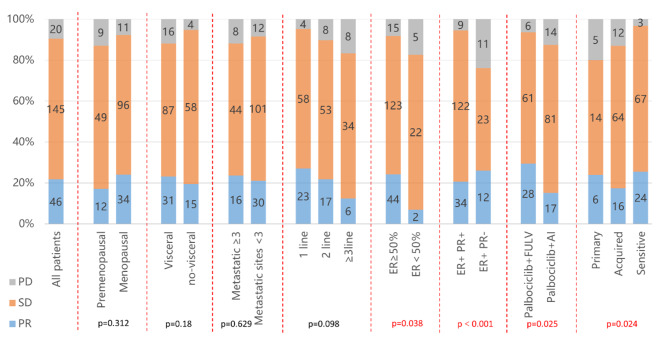
Best response to Palbociclib plus endocrine therapy of patients with different characteristics P-values of < 0.05 indicate statistical significance and are marked in red. ER, Estrogen receptor; FULV, Fulvestrant; AI, Aromatase Inhibitors; PR, Partial response; SD, Stable disease; PD, Progressive disease

For the best response, 46 (21.8%) patients achieved PR, 145 (68.7%) patients showed SD (Table 3), and 20 (9.5%) patients developed PD, with an ORR of 21.8% and a DCR of 90.5% (Table 3). Regarding treatment lines with Palbociclib, the ORR and DCR was 27.1% and 95.3% in patients treated as first-line, both higher than that in those treated as second line (ORR: 21.8%, DCR: 89.7%) and third or later line (ORR: 12.5%, DCR: 83.3%). The 6- and 12-month PFS rates of total population were 64.5% and 30.8%, respectively. Favorable tumor response was shown in patients with high ER expression (P = 0.038), Luminal A type (P < 0.001) and received concomitant fulvestrant (P = 0.025). Besides, sensitivity to the most recent ET was associated with the response to Palbociclib plus ET (P = 0.024) (Fig. 2).

**Table 3:** Effectiveness of Palbociclib-based treatment in different treatment-line settings

	**All patients** **(n = 211)**	**1st line** **(n = 85)**	**2nd line** **(n = 78)**	**≥ 3rd line** **(n = 48)**
**Best response**				
Partial response	46(21.8)	23(27.1)	17(21.8)	6(12.5)
Stable disease	145(68.7)	58(68.2)	53(67.9)	34(70.8)
Progressive disease	20(9.5)	4(4.7)	8(10.3)	8(16.7)
**Objective response rate**	46(21.8)	23(27.1)	17(21.8)	6(12.5)
**Disease control rate**	191(90.5)	81(95.3)	70(89.7)	40(83.3)
**Progression-free rate**				
6 months	136(64.5)	59(69.4)	43(55.1)	34(70.8)
12 months	65(30.8)	33(38.8)	21(26.9)	11(22.9)
**Survival outcome (months)**				
Median PFS (95% CI)	12.2(10.1–14.3)	14.5(11.1–17.9)	10.6(4.1–17.1)	8.7(6.8–10.7)

Values are presented as number (%) unless otherwise indicated. CI, confidence interval

**Fig. 3:**
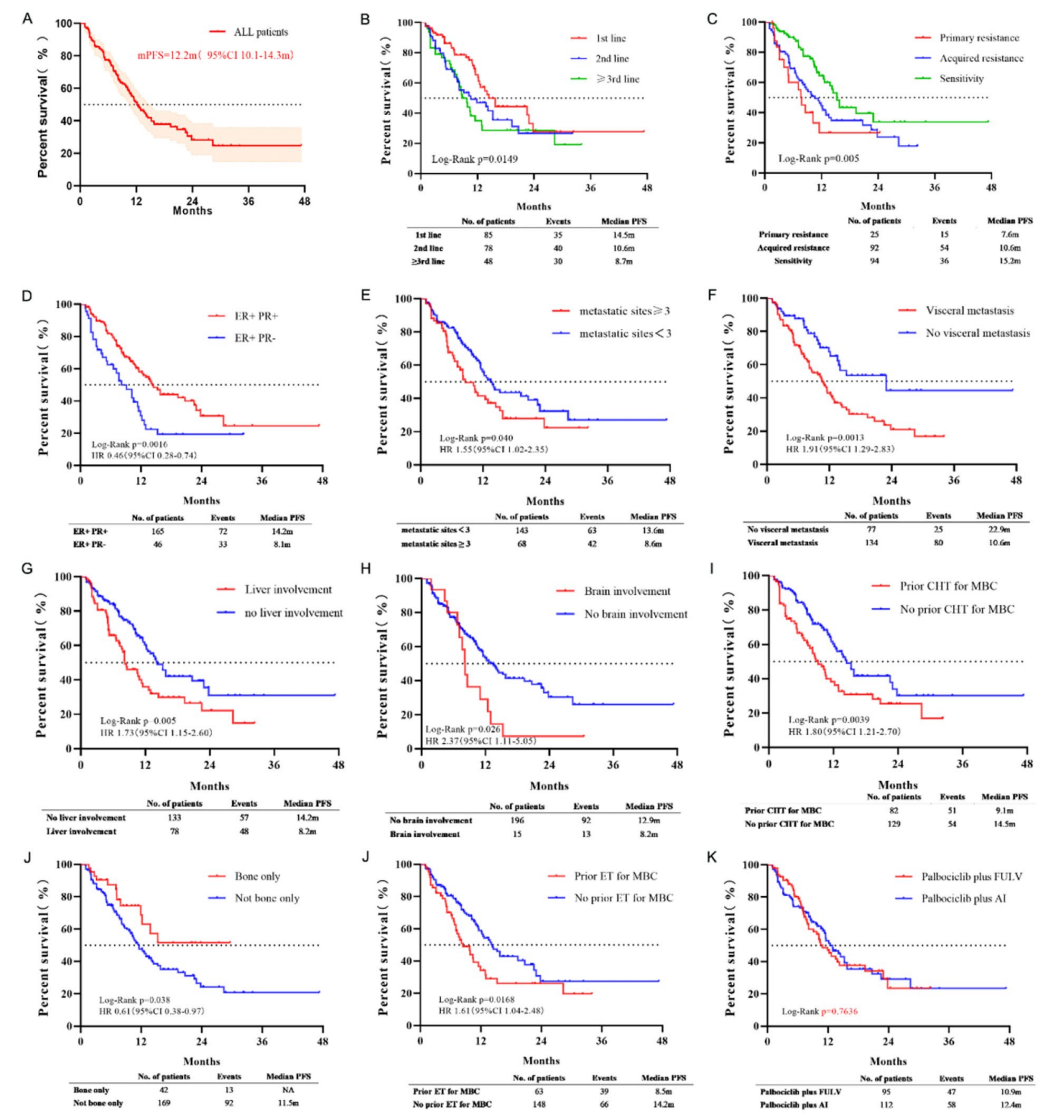
Progression-free survival of Palbociclib plus endocrine therapy stratified by patient characteristics. (A) All patients; (B) Treatment-line settings; (C) Sensitivity to the most recent ET; (D)Performance status; (E) Number of metastatic sites; (F) Presence of visceral metastasis; (G) Metastatic tumors involving liver; (H) Metastatic tumors involving brain; (I) Only bone metastases; (J) Whether or not receiving chemotherapy for MBC; (K) Whether or not receiving ET for MBC; (L) Type of concomitant ET. Kaplan-Meier method and compared by the log-rank test. P-values of less than 0.05 indicate statistical significance. PFS, Progression-free survival; HR, Hazard ratio; CI, Confidence interval; NA, Not available; CHT, Chemotherapy; MBC, Metastatic breast cancer; ET, Endocrine therapy; FULV, Fulvestrant; AI, Aromatase Inhibitors

At the time of survival data extraction, 106 patients were still receiving Palbociclib-based treatment. After a median follow-up period of 14.2 months (range, 2.1 to 47.3 months), the median PFS of the overall population was 12.2 months (95% CI: 10.1–14.3 months), and the median OS was not reached. The median PFS was 14.5 months, 10.6 months and 8.7 months in patients received Palbociclib in first-, second-, and third- or later-line setting, respectively (Fig. 3).

**Fig. 4:**
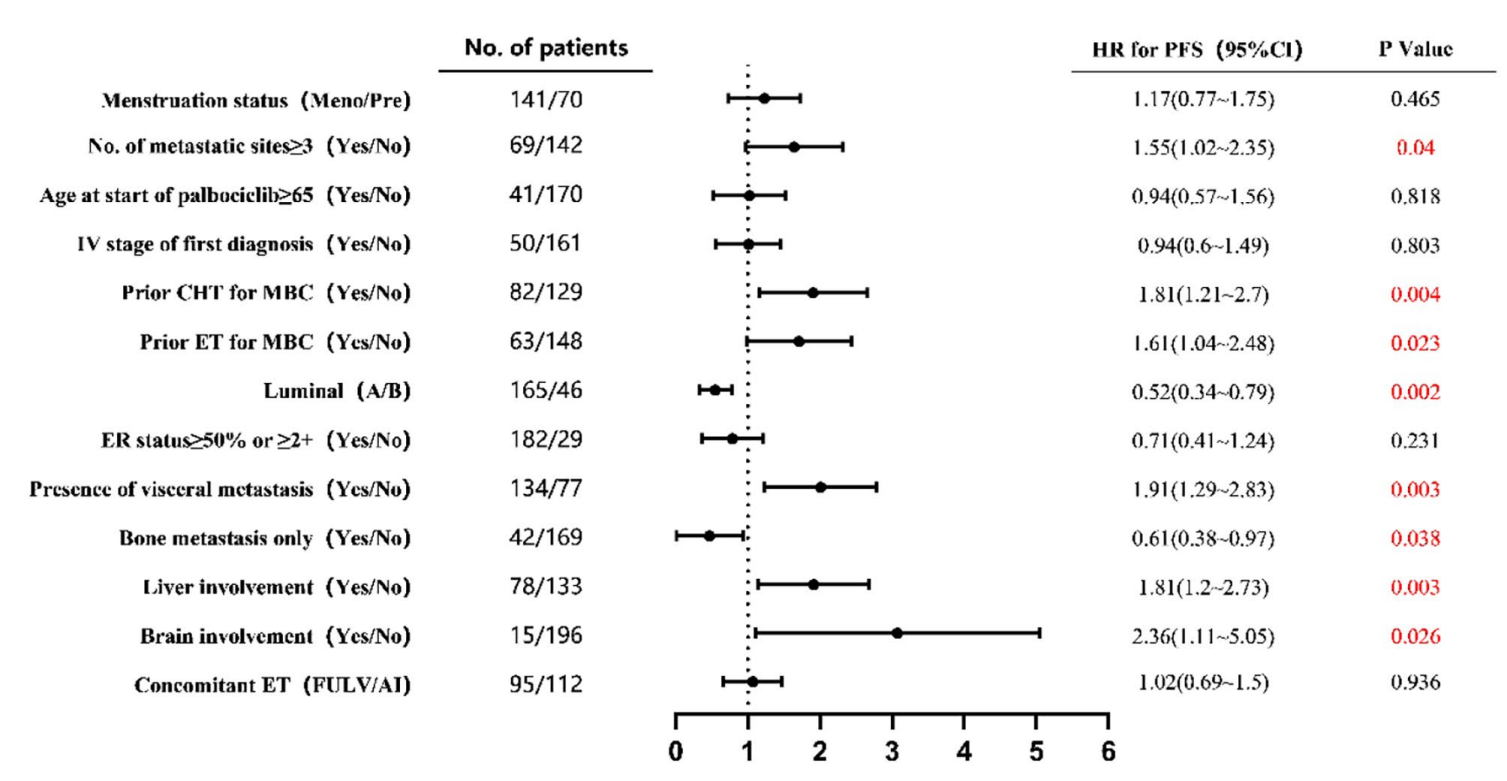
Forest plot of post-hoc subgroup analysis. HR, Hazard ratio; CI, Confidence interval; Meno, Menopausal status; Pre, Premenopausal status; CHT, Chemotherapy; MBC, Metastatic breast cancer; ET, Endocrine therapy; ER, Estrogen receptor; FULV, Fulvestrant; AI, Aromatase Inhibitors

More than three metastatic sites (HR: 1.55, 95% CI: 1.02 to 2.35, P = 0.04), with visceral metastasis (HR: 1.91, 95% CI: 1.29 to 2.83, P = 0.003), with liver metastasis (HR: 1.81, 95% CI: 1.20 to 2.73, P = 0.003), with brain metastasis (HR: 2.36, 95% CI: 1.11 to 5.05, P = 0.026), prior chemotherapy (HR: 1.81, 95% CI: 1.21 to 2.70, P = 0.004), and prior ET (HR: 1.61, 95% CI: 1.04 to 2.48, P = 0.023) were associated with a worse PFS, while patients with Luminal A type MBC (HR: 0.52, 95%CI: 0.34 to 0.79, P = 0.002) and with bone metastasis only (HR: 0.61, 95% CI: 0.38 to 0.97, P = 0.038) showed a prolonged PFS (Fig. 4).

### Safety profiles

The most common AE was hematological toxicity; 141 (66.8%) patients experienced neutropenia, with 77 (36.5%) displaying grade 3–4 neutropenia (Table 4). Anemia and thrombocytopenia of any grade were reported in 23 (10.9%) and 30 (14.2%) of patients, respectively, with grade 1–2 predominating. The treatment-related non-hematological toxicity was manageable, with the most frequent AEs being asthenia, alopecia, and nausea/vomiting (23 (10.9%), 11 (5.2%), and 10 (4.7%), respectively). Besides, few patients developed blood biochemical abnormalities.

**Table 4:** Treatment-related adverse events

**Adverse events**	**Any grade**	**Grade 1**	**Grade 2**	**Grade 3**	**Grade 4**
**Hematologic**					
Anemia	23(10.9)	14(6.6)	7(3.3)	1(0.5)	1(0.5)
Leukopenia	151(71.6)	15(7.1)	87(41.2)	47(22.3)	2(0.9)
Neutropenia	141(66.8)	18(8.5)	46(21.8)	57(27.0)	20(9.5)
Thrombocytopenia	30(14.2)	11(5.2)	14(6.6)	3(1.4)	2(0.9)
**Non-hematologic**					
Nausea/vomiting	10(4.7)	10(4.7)	-	-	-
Diarrhoea	4(1.9)	3(1.4)	1(0.5)	-	-
Anorexia	6(2.8)	5(2.4)	1(0.5)	-	-
Dry eyes/Blurred vision	3(1.4)	3(1.4)	-	-	-
Skin rash	6(2.8)	6(2.8)	-	-	-
Mucositis	8(3.8)	8(3.8)	-	-	-
Alopecia	11(5.2)	11(5.2)	-	-	-
Fatigue	23(10.9)	19(9.0)	4(1.9)	-	-
**Blood biochemical**					
Elevated TBIL	4(1.9)	1(0.5)	1(0.5)	-	2(0.9)
Elevated ALT/AST	10(4.7)	6(2.8)	1(0.5)	1(0.5)	2(0.9)
Decreased ALB	1(0.5)	-	-	1(0.5)	-

Values are presented as number (%) unless otherwise indicated. TBIL, Total bilirubin; ALT, alanine aminotransferase; AST, Aspartate Aminotransferase; ALB, Albumin

A total of 181 (85.8%) patients started Palbociclib at 125 mg/day, and 21 (10.0%) patients reduced to 100 mg/day. 29 (13.7%) patients started Palbociclib at 100 mg/day, and 11 (5.2%) patients reduced to 75 mg/day. Only 1 (0.5%) patient started Palbociclib at 75 mg/day. The rate of AE leading to dose reduction was 10%, and no patient experienced AE leading to treatment discontinuation (Table 5).

**Table 5:** Dose of Palbociclib-based treatment

**Start dose of Palbociclib (mg/day)**	
* Subtotal*	211(100)
Start at 125	181(85.8)
Start at 100	29(13.7)
Start at 75	1(0.5)
**Dose reduction (mg/day)**	
* Subtotal*	32(15.2)
From 125 to 100	21(10.0)
From 100 to 75	11(5.2)

Values are presented as number (%) unless otherwise indicated

## Discussion

Overall, patients in our study who received Palbociclib plus ET as the first-line treatment for HR+/HER2- advanced or MBC had a median PFS of 12.2 months, which matched the 12 months from other retrospective studies [21, 22], indicating similar benefits for Palbociclib in treating patients across different countries, races and health care systems. Although the ORR was only 21.8%, the DCR reached 90.5%. With median PFS of 14.2, 10.6 and 8.7 months in the first-, second- and later-lines, the data provided strong support for earlier initiation of Palbociclib. In this study, Palbociclib-based therapy was shown to benefit MBC patients even with later lines of therapy, indicating that Palbociclib plus ET is a viable treatment option for patients with HR+/HER2- MBC in China.This study differed from some previously published studies. Treatment lines of Palbociclib was an important factor associated with patient outcomes. Previous real-world studies with a majority of MBC patients received Palbociclib plus ET as the first-line treatment have showed a median PFS of 20 months or longer [14, 16, 23, 24] or a DCR of more than 90% [25, 26]. Besides, in studies with patients mostly previously heavily treated and initialed Palbociclib-based therapy in the third or later lines, the PFS was only 6 to 8 months [17, 22, 27, 28]. For studies included all patients treated with Palbociclib-based therapy as first-line, the second-line and later-line, the outcomes were comparable to ours, with a median PFS of 10 to 12 months[13, 21, 29]. In Northwest area of China, being limited by conditions such as economic income, medical insurance reimbursement, etc., patients with MBC commonly preferred traditional chemotherapy to novel treatments. In this study, the percentage of patients declined from 40 to 37% and 23%, respectively, for the first-line, second-line, and third or later-line treatments. In the subgroup of patients who had progressed on prior ET, a Palbociclib-based second-line treatment resulted in similar response rates to PALOMA3, with ORRs of 21.8% vs. 19%, and clinical benefit rates (CBRs) of 89% vs. 80%, respectively. A slight difference was observed between our results of second-line and PALOMA3 trial (10.6 vs. 11.2 months), which may due to the heterogeneity of study population, with more patients being frail or having previously been heavily treated in our study. In our study, we had an increased number of PS more than 2 (3.8% vs. 0%), more patients with visceral metastasis (66% vs. 59%), and more patients receiving prior chemotherapy as (neo)adjuvant therapy (62% vs. 40%) as well as metastatic setting (48.7% vs. 33%). Studies such as these can add valuable data to the evidence of published studies, especially for patients underrepresented in clinical trials.

Based on clinical records, we identified the role of Palbociclib in MBC patients in the Northwest of China. The differences between the real-world and clinical studies may have affected therapeutic results even though they cannot be directly compared due to different study designs and patient populations (Supplement Table 1) [8, 9]. According to Yuan et al., more than two thirds of Chinese patients with HR+/HER2- MBC received chemotherapy in addition to ET as the first-line treatment [30]. Approximately 40% of patients received prior chemotherapy for metastatic disease and 3.5% of patients had poor PS in this study, both representing substantial portions of the population in clinical practice but not eligible for clinical trials. Therefore, this study’s worse PFS may be due to the inclusion of such advanced or heavily pretreated patients than that observed in clinical trials and other studies. The update OS analysis of PALOMA 3 revealed that subpopulations who received prior chemotherapy for MBC benefit less from Palbociclib plus ET [31]. In our study, patients with no prior chemotherapy for MBC had a better PFS than those who had prior chemotherapy (HR = 0.55; P = 0.004), as did those who had received no prior endocrine therapy for MBC (HR = 0.62; P = 0.023). Palbociclib-based therapy was then challenged as one choice of maintenance therapy. While the PALOMA studies included approximately 15–20% Asians, our cohort was six years younger (with median age of 55 and 51 years in the first-line and second-line subgroup, respectively) than those in the PALOMA-2 (median age 62) and PALOMA-3 (median age 57) trials. In addition, patients under 65 years old were more prevalent in our study than in the PALOMA study (78% vs. 41%). A young age is associated with poor survival among breast cancer patients [32], which may also contribute to the inferior outcome.

Patients receiving Palbociclib-based therapy as the first-line treatment in our study achieved a DCR of 95% that was comparable to the CBR of 85% in the PALOMA 2 trial. This may be due to the different definition of DCR in the PALOMA2 trial, where it was defined as a disease that was stable for at least 24 weeks, whereas there was no time restriction for SD in our study. Nevertheless, the ORR was significantly lower in our study than that in PALOMA2 trial (27% vs. 42%). In addition to the younger median age mentioned above, the main reason is the inclusion of more premenopausal women (31% vs. 0%), and the higher percent of patients who had already received prior (neo)adjuvant chemotherapy (70.6% vs. 47.5%) as well as prior palliative chemotherapy (8.2% vs. 0%) in comparison to the Asian subgroup from PALOMA2. Especially, the 8.2% of patients who received prior chemotherapy for metastatic disease tend to have heavy tumor burden or visceral crisis. Their inclusion in our study may result in a lower response rate and shorter PFS. In previous clinical trials focused on Palbociclib, premenopausal women with breast cancer were under-represented. Further analysis of the POLAMA3 results revealed that Palbociclib combined with fulvestrant improved the PFS for premenopausal women with prior endocrine-resistant HR+/HER2- MBC (9.5 versus 5.6months, respectively) (HR = 0.50, 95% CI: 0.29–0.87) [33]. Premenopausal women in the PALOMA3 trial had an ORR of 25.6% and a CBR of 69.4%, comparable to the ORR of 17.1% and DCR of 87.1% in this study. Both results showed no significant difference in efficacy regardless of the menopausal status of the patients (P = 0.312). Recently, the DAWNA-2 study, a Chinese multicenter phase 3 trial, reported that Dalpiciclib (a CDK4/6 inhibitor) significantly improved PFS in patients with HR+/HER2- advanced breast cancer compared to placebo when combined with letrozole or anastrozole as first-line therapy (mPFS 30.6 m vs. 18.2 m, HR = 0.51, p < 0.0001) [34]. The study included a high proportion of patients with visceral metastases (60.7%) and premenopausal/menopausal population (38.2%), reflecting the characteristics of the Chinese population. Such an RCT also showed no significant difference between the premenopausal and postmenopausal subgroups, which is consistent with our findings.

All these data reflected a high proportion of “high risk” patients in our study. Another reason for lower ORR was the inclusion of more patients with only bone metastases (23.5% vs. 18.5%), where the response evaluation could be either SD or PD according to RECISTv1.1. As well as the population composition, the difference may be the result of not requesting radiological confirmation in response judgement. As a result of the above factors, Palbociclib-based first-line therapy has a shorter median PFS of 14.5 months in comparison to the 24.8 months reported in PALOMA2 study.

Due to the assumption that ET sensitivity was associated with CDK4/6i response, several studies on Palbociclib were conducted primarily on MBC patients who were sensitive to ET [35, 36]. Palbociclib plus ET, however, also showed superior efficacy and improved life quality among patients resistant to ET [37, 38]. Compared to PALOMA trials, our study seemed to include less patients sensitive to ET (44.5% vs. 79%). However, the definition of endocrine sensitivity was not standardized worldwide at the beginning of the PALOMA trial, in which patients with a documented clinical benefit from at least one previous ET in the metastatic setting or treatment with at least 24 months of adjuvant ET before disease progression were identified as endocrine sensitive, otherwise they were defined as ET resistant. Nevertheless, our study considered patients to be ET-sensitive if they had untreated stage IV disease or relapsed at least one year after withdrawal of adjuvant ET, according to ESMO recommendation. There is a need to clarify that patients with de novo stage IV disease were ET naïve, not equal to ET sensitive, and some of them proved to be endocrine resistant in the subsequent treatment. In contrast, our study included fewer patients with disease free survival less than 24 months during adjuvant ET (11.8% in our study, 18% in PALOMA3 and more than 20% in PALOMA2), which would suggest less patients with primary resistance. Despite this, we could infer that Palbociclib-based therapy in Asian patients was not inferior in efficacy to that of western populations.

Most previous real-world studies did not report the endocrine sensitivity status of patients. Only three studies reported the percentage of patients with endocrine resistance, with rates of 9%, 32% and 80%, respectively [12, 15, 16]. A study in China distinguished primary resistance from acquired resistance, with each accounting for 37.7% and 43.1% of cases respectively [15]. However, their final survival analysis combined patients with sensitivity and those with acquired resistance into one group, then further compared to those with primary resistance. This real-world study was first to confirm that patients with endocrine sensitivity have the best prognosis when receiving Palbociclib plus ET, followed by patients with acquired resistance and then patients with primary resistance. Moreover, these findings provide evidence for CDK4/6i application in patients with different responses to ET. The addition of Palbociclib to ET may improve the efficacy of endocrine sensitive patients to some extent. In addition, it reversed their resistance to ET among patients with acquired resistance. However, the benefits of Palbociclib for patients with primary resistance to ET remain uncertain. Although primary and acquired resistance are currently differentiated based on the response duration, studies have revealed distinct genes involved in the two kinds of resistance [39].

ET sensitivity is closely related to the expression levels of hormone receptors, as was the efficacy of Palbociclib. Studies have shown a controversial association between PgR levels and benefit from CDK4/6i in breast cancer. PALOMA3 found a positive correlation between high PgR levels and superior outcomes in patients receiving either Palbociclib or placebo when combined with fulvestrant. Michela et al. found that PgR positivity had no significant impact on the PFS of 71 MBC patients receiving CDK4/6i when the PgR-positivity cutoff was set at ≥ 1% immunoreactive cells [40]. In addition, Shao et al. found that PgR ≥ 20% was associated with longer PFS (8.5 vs. 6.7 months) without significance (P = 0.08) in 81 cases. The change in PgR levels from primary to metastatic lesions was related to PFS [41]. PFS was longer for patients whose PgR remained high or changed from low to high than for those whose PgR remained low or changed from high to low. Palbociclib-based therapy resulted in a higher survival rate for patients with double-positive ER and PgR than for those with ER single positive disease in our study. The widely-used diagnosis kit and mature detective technology have made PgR a highly reproducible assay for breast cancer subtypes. Our results showed that low PgR expression negatively impacted PFS.

A meta-analysis of three RCT studies revealed that addition of CDK4/6i to ET significantly improved OS among patients with at least three metastatic sites (HR = 0.75, P = 0.02) [42]. In this study, the PFS benefit was also observed in patients with fewer than three metastatic sites (HR = 1.55, P = 0.04). Among patients with bone metastasis only, Yuan et al. found that ET was preferred as their first-line treatment [30], and further study by Schettini et al. showed that the addition of CDK4/6i to ET had a non-statistically significant benefit (HR = 0.82, P = 0.23) [42]. However, our study found that patients with bone metastasis only had better PFS than patients with metastasis to other organs (HR = 0.61; P = 0.038), especially to brain or to liver.

This study represents, to our knowledge, the largest Chinese cohort treated with Palbociclib in an unselected real-world setting, providing a considerable amount of data in support of the efficacy and tolerability of Palbociclib. However, some limitations should be acknowledged. As data were only collected by physicians willing to participate in the study, there was a potential for selection bias; however, physicians were asked to select consecutive patients in accordance with the index date. Other limitations were inherent to the observational retrospective study design. These included missing information about some baseline characteristics, laboratory data, and incomplete documentation about treatment toxicities. Furthermore, there is no uniform schedule or interval for imaging evaluation. Additionally, our follow-up period is relatively short, and further analysis of the data will be needed after a longer period of follow-up. As a result, direct comparison with other clinical studies may be difficult.

## Conclusion

Palbociclib plus endocrine therapy exhibited favorable effectiveness and manageable toxicities in the real-world setting, supporting their use in Chinese patients with HR+/HER2 − MBC. Safety of the drugs was comparable to the previous pivotal trials, no new safety signal was reported, and toxicity was manageable. Further studies are required to provide mature outcome data.

## Electronic supplementary material

Below is the link to the electronic supplementary material.

Supplementary Material 1

## Data Availability

Data and materials were identified from the medical records as the methods section. The datasets analyzed during the current study are available from the corresponding author on reasonable request.

## List of abbreviations

ABC5: the 5th International Consensus Conference for Advanced Breast Cancer
AE: adverse events
AI: aromatase inhibitors
AJCC TNM: American Joint Committee on Cancer tumor-node-metastasis
CBR: clinical benefit rate
CDK4/6i: cyclin-dependent kinase 4/6 inhibitors
CI: confidence interval
CR: complete response
CTCAE 4.0: Common Terminology Criteria for Adverse Events version 4.0
DCR: disease control rate
ECOG PS: Eastern Cooperative Oncology Group performance status
ER: estrogen receptor
ET: endocrine therapy
HR: hazard ratios
HR+/HER2-: hormone receptor positive and human epidermal growth factor receptor 2 negative
IHC: immunohistochemistry
MBC: metastatic breast cancer
ORR: objective response rate
PR: partial response
PgR: progesterone receptor
PFS: progression-free survival
RECISTv1.1: Response Evaluation Criteria in Solid Tumors version 1.1
SERD: selective estrogen receptor degrader
SERM: selective estrogen receptor modulators
SD: stable disease
