# Supplementary material for: Palbociclib plus endocrine therapy in hormone receptor-positive and HER2 negative metastatic breast cancer: a multicenter real-world study in the northwest of China
Source: BMC Cancer. 2023 Jan 30;23:103. doi: 10.1186/s12885-023-10568-0 (PMC9885675; doi:10.1186/s12885-023-10568-0)
Supplement: Supplementary file 1 — Supplementary Material 1 [file 12885_2023_10568_MOESM1_ESM.pdf]

Table S1. Data comparison between the current study and PALOMA trials.

	The current study			PALOMA2		PALOMA3
**Subgroup**	All	1st line	2d line	Asian	Non-Asian	All
**Sample size (n)**	211	85	78	65	379	347
**Treatment**						
**Pal+FULV**	53.1%	41.2%	55.1%	0%	0%	100%
**Pal+AI**	45.0%	55.3%	43.6%	100%	100%	0%
**Age (median, range)**	53 (29–88)	55 (29-84)	52(32-88)	61(43-88)	62(30-89)	57(30–88)
**≥65y**	19.4%	21.2%	21.8%	58.5%	59.4%	
**＜65y**	80.6%	78.8%	78.2%	41.5%	40.6%	
**ECOG PS**						
**0**	34.6%	34.1%	38.5%	60%;	57.5%;	59%;
**1**	61.6%	62.4%	57.7%	30.8%	41.7%;	41%
**2**	3.8%	3.5%	3.8%	9.2%	0.8%	0
**Race**						
**The rate of Asian**	100%	100%	100%	14.6%		21.0%
**Menopausal Status**						
**Menopausal**	67%;	69%;	68%	100%		79%
**Premenopausal**	33%	31%	32%	0%		21%
**Initial stage**						
**De novo stage IV**	23.7%	31.8%	15.4%	20%	33%	-
**Relapsed ⩽12m**				20%		
**Metastatic site**						
**Bone only**	19.9%	23.5%	20.5%	18.5%	24.0%	22%
**Visceral**	63.5%	51.8%	66.7%	53.8%	47.2%	59%
**Prior Neo(adjuvant) therapy**	73.0%	70.6%	66.7%	50.8%	47.5%	40%
**CHT**	60.7%	52.9%	38.5%	95.5%	75%	21%
**ET**						
**Prior therapy for MBC**						
**CHT**	38.9%	8.2%	48.7%	0%	0%	33%
**ET**	29.9%	0%	38.5%	0%	0%	46%
**PFS (m)**	12.2	14.5	10.6	25.7	27.6	11.2
**CR**	0%	0%	0%	1.5%	2.4%	0%
**ORR**	21.8%	27.1%	21.8%	49.2%	45.9%	19%
**DCR**	90.5%	95.3%	89.7%	84.6% ‡	86.0% ‡	80%

‡ CR + PR + SD ≥ 24 weeks.

PAL, palbociclib; FULV, Fulvestrant; AI, Aromatase Inhibitors; ECOG, Eastern Cooperative Oncology Group; CHT, chemotherapy; ET, Endocrine therapy; PFS, Progression-free survival; CR, Complete

response; PR, partial response; SD, stable disease; ORR, objective response rate; DCR, disease control rate.
